# Tracheal tube and laryngeal mask cuff pressure during anaesthesia - mandatory monitoring is in need

**DOI:** 10.1186/1471-2253-10-20

**Published:** 2010-12-03

**Authors:** Kim Z Rokamp, Niels H Secher, Ann M Møller, Henning B Nielsen

**Affiliations:** 1 Department of Anaesthesia, University of Copenhagen, Herlev Hospital, Herlev Ringvej 7, DK-2730 Herlev, Denmark; 2 Department of Anaesthesia, University of Copenhagen, Rigshospitalet, Blegdamsvej 9, DK-2100 Copenhagen, Denmark

## Abstract

**Background:**

To prevent endothelium and nerve lesions, tracheal tube and laryngeal mask cuff pressure is to be maintained at a low level and yet be high enough to secure air sealing.

**Method:**

In a prospective quality-control study, 201 patients undergoing surgery during anaesthesia (without the use of nitrous oxide) were included for determination of the cuff pressure of the tracheal tubes and laryngeal masks.

**Results:**

In the 119 patients provided with a tracheal tube, the median cuff pressure was 30 (range 8 - 100) cm H_2_O and the pressure exceeded 30 cm H_2_O (upper recommended level) for 54 patients. In the 82 patients provided with a laryngeal mask, the cuff pressure was 95 (10 - 121) cm H_2_O and above 60 cm H_2_O (upper recommended level) for 56 patients and in 34 of these patients, the pressure exceeded the upper cuff gauge limit (120 cm H_2_O). There was no association between cuff pressure and age, body mass index, type of surgery, or time from induction of anaesthesia to the time the cuff pressure was measured.

**Conclusion:**

For maintenance of epithelia flow and nerve function and at the same time secure air sealing, this evaluation indicates that the cuff pressure needs to be checked as part of the procedures involved in induction of anaesthesia and eventually checked during surgery.

## Background

During general anaesthesia, pulmonary ventilation is secured with a tracheal tube or by a laryngeal mask and attention to the risk of complications related to a high intracuff pressure is important. When the cuff to tracheal wall pressure exceeds the tracheal capillary pressure (27-40 cm H_2_O) for approximately 15 min, the tracheal mucous membrane becomes ischemic [1]. The intracuff pressure approximates the cuff to tracheal wall pressures in high volume/low pressure cuffs [2] and a cuff pressure below 30 cm H_2_O is recommended to prevent ischemic injury [1,3]. Also recurrent laryngeal nerve palsy has been demonstrated in up to 5% of patients after intubation and a high cuff pressure is suspected to be important in that regard [4,5]. Similarly in patients provided with a laryngeal mask, a high cuff pressure may lead to palsy of the lingual, hypoglossal, and recurrent laryngeal nerves [6-8] but with the cuff pressure maintained below 60 cm H_2_O, the airway seal is optimized [9,10] and the incidence of a postoperative sore throat is low [11-13].

Here patients, provided with either a tracheal tube or a laryngeal mask during elective surgery requiring general anaesthesia, were assessed for the established cuff pressure. We aimed at evaluating the incidence of a cuff pressure that was outside the recommended level and hypothesized that especially overweight patients would be exposed to a high cuff pressure since a high body mass index caries a risk of gastroesophageal reflux [14] and that such patients require a high peak inspiratory pressure during mechanical ventilation [15].

## Methods

The study was evaluated by The Scientific Ethics Committee of the Capital Region of Denmark (Journal no. H-4-2010-075) but not considered to require ethical approval since it was directed to quality control and did not involve any experimental procedures. We determined the cuff pressure in consecutetively enrolled patients from two hospitals: 97 from Herlev Hospital and 104 from Rigshospitalet. We included adult patients planned for operation in general anaesthesia and who were provided either with a tracheal tube or a laryngeal mask, while we excluded patients who had been intubated prior to arriving at the operating room and those for whom the airway was kept patent with a double-lumen tracheal tube.

When the patient arrived at the operating ward, the staff prepared the patient for anaesthesia and surgery according to local instructions and the anaesthesiologist in charge of the patient initiated the anaesthesia together with an anaesthesia nurse. For ventilatory and cardiovascular monitoring a Dräger CATO (type M32040, Lübeck, Germany) or Dräger Primus (G18155) anaesthesia machine was used together with a Phillips Intillivue MP70 monitor. Neuromuscular function was evaluated with a TOF monitor (Organon Dublin, Ireland). For induction of anaesthesia propofol or thiopental was administered guided by the weight of the patient and the administration was continued until the cilia reflex was eliminated. Anaesthesia was maintained with propofol, sevoflurane or desflurane and fentanyl or remifentanil was used for analgesia, while cisatracurium, rocuronium or suxamethonium facilitated tracheal intubation. The airway was kept patent with a tracheal tube (high volume/low pressure; Unomedical, Copenhagen, Denmark; n = 119) or a laryngeal mask (AuraOnce; Ambu A/S, Ballerup, Denmark; n = 82) for mamma (n = 49), gastrointestinal (n = 33), gynaecological (n = 11), orthopaedic (n = 5), plastic (n = 11), urological (n = 71), hepatic (n = 6), or vascular surgery (n = 15). Inflation of the cuff was not described in the local instructions for induction of anaesthesia and therefore carried out according to the disposition of the anaesthesiologist in charge of the patient and without the use of a manometer or a pressure release valve. In regard to this study there were no restrictions to the treatment of the patient before or after the cuff pressure was measured. According to local tradition, the airway cuff pressure is not adjusted during the surgical intervention and manipulation of the head does not regularly provoke a recheck of the cuff pressure.

When anaesthesia was established and the tracheal tube or the laryngeal mask was in place, the cuff pressure was determined by a Universal cuff pressure gauge with an upper scale limit of 120 cm H_2_O (VBM Medizintechnik GmbH, Baden-Württemberg, Germany). The manometer was connected to the pilot balloon and the cuff pressure was read from the manometer and documented together with the height, weight, age, and sex of the patient and the type of surgery and the time from placement of the tube or mask to when the cuff pressure was measured. In case the cuff pressure was outside the recommended level [3,12], the pressure was adjusted.

Considering that we should detect 10 patients provided with a too high laryngeal or tracheal tube pressure in each department and the incidence is about 25% [16], we evaluated the cuff pressure in 200 patients. Data are presented as median and range and a p-value < 0.05 was considered to be statistical significant. For correlations between cuff pressure and age, body mass index, type of surgery, and time from induction of anaesthesia to determination of the cuff pressure, Pearson's correlation was used.

## Results

**Figure 1:**
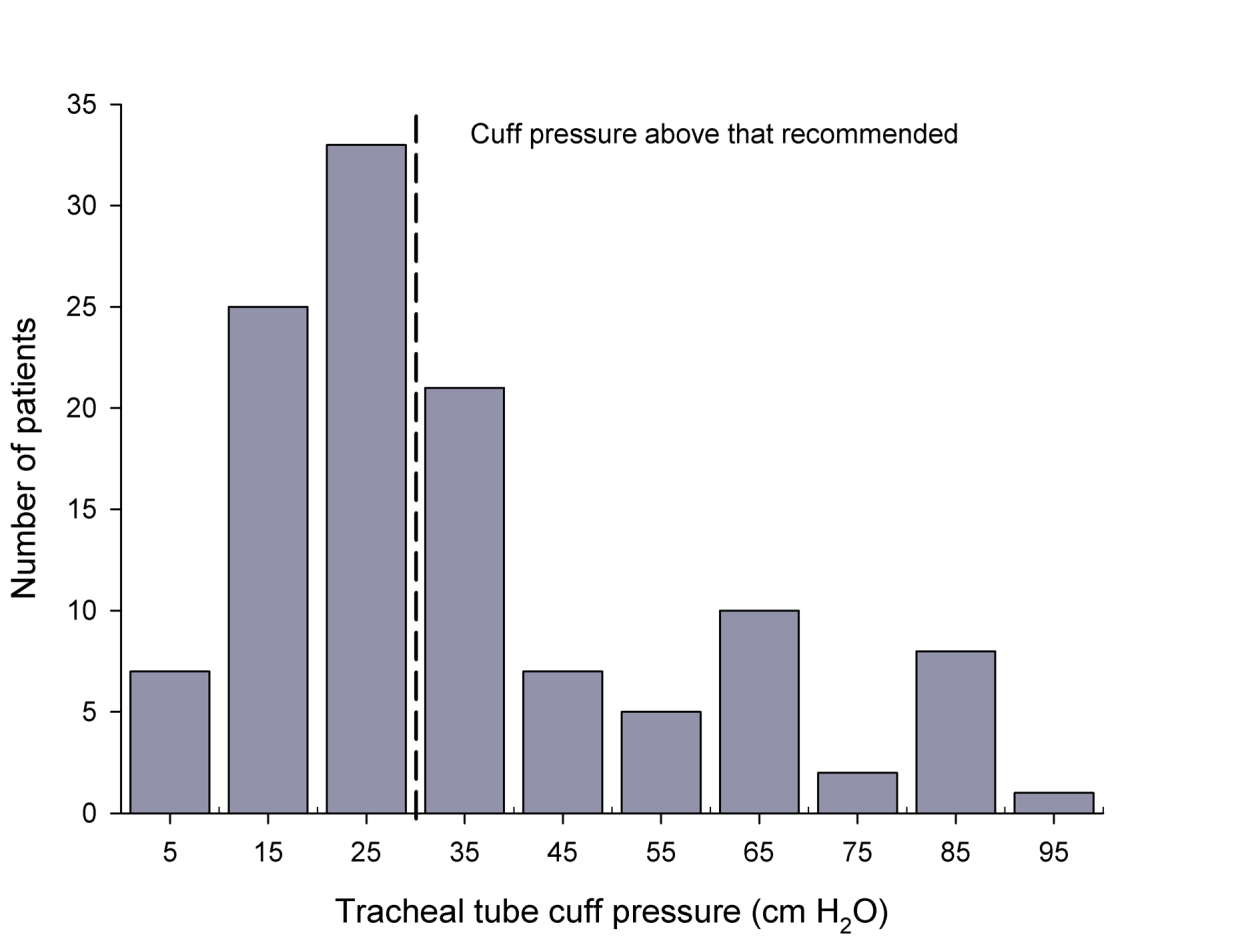
**Patients provided with different tracheal tube cuff pressures**. Broken line indicates the upper recommended level.

We determined the cuff pressure in 110 female and 91 male patients, age 61 (18 - 93) years and body mass index 24.6 (14 - 48) kg m^-2 ^and the cuff pressure was determined 58 (2 - 360) min after induction of anaesthesia. In the 119 patients provided with a tracheal tube during surgery, the cuff pressure was 30 (8 - 100) cm H_2_O and it exceeded 30 cm H_2_O for 54 patients, and the pressure was higher than 40 cm H_2_O for 33 patients (Figure 1) with no significant difference between values obtained in the two departments.

**Figure 2:**
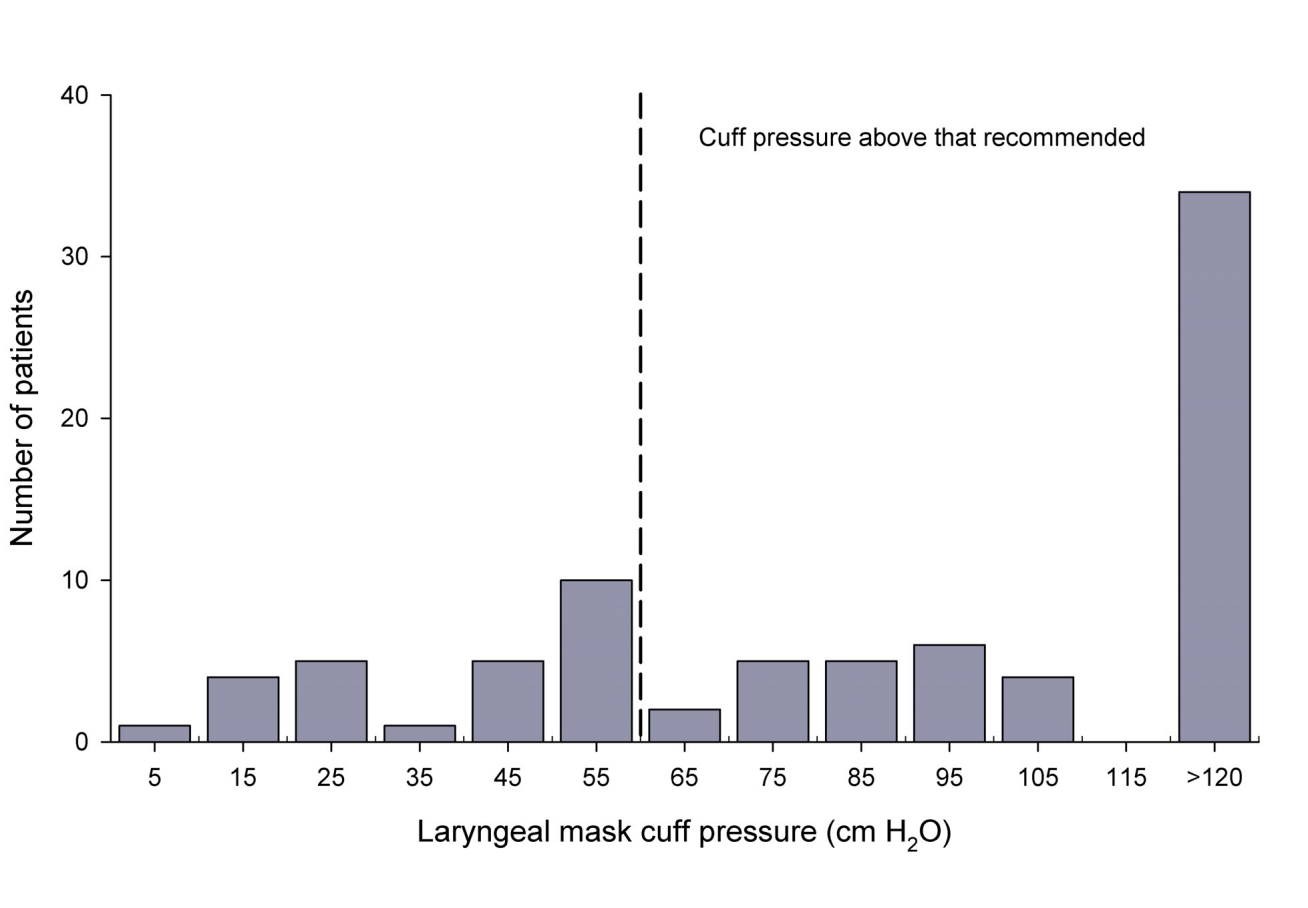
**Patients provided with different laryngeal mask cuff pressures**. Broken line indicates the upper recommended level.

For the 82 patients provided with a laryngeal mask, the cuff pressure was 95 (10 - 121) cm H_2_O reflecting that for 56 patients, the cuff pressure was above 60 cm H_2_O and it exceeded the upper gauge limit for 34 patients (Figure 2) and also for the established laryngeal mask cuff pressure, there were no significant difference between values obtained in the two departments.

There was no significant relation between tracheal tube cuff pressure and age (r = 0.028), body mass index (r = 0.245), type of surgery (r = -0.001), or the time from induction of anaesthesia to determination of the cuff pressure (r = -0.168). Furthermore there was no significant relation between laryngeal mask cuff pressure and age (r = 0.129), body mass index (r = -0.015), type of surgery (r = -0.177), or the time from induction of anaesthesia to when the cuff pressure was determined (r = -0.074).

## Discussion and Conclusion

In contrast to what we expected, overweight patients were not more frequently exposed to a high cuff pressure than other patients but even without the use of nitrous oxide for maintained anaesthesia, the cuff pressure exceeded the recommended level for about half of the patients provided with a tracheal tube and for almost three quarters of those patients provided with a laryngeal mask. We expected a high cuff pressure to be predominant in obese patients since a high body mass index caries a risk of gastroesophageal reflux [14] and a high peak inspiratory pressure during mechanical ventilation [15]. A low laryngeal mask cuff pressure secures the airway seal [11,10] and reduces the incidence of postoperative sore throat [11]. It was therefore surprising to us that the majority of the patients were exposed to a too high cuff pressure and the most frequently measured laryngeal cuff pressure was in fact at a level that exceeded 120 cm H_2_O, arguing for that cuff pressure is an underestimated issue in general anaesthesia practice.

Sengupta et al. [16] found that among 93 patients provided with a tracheal tube and undergoing general anaesthesia, 50% of the patients were having a cuff pressure above 30 cm H_2_O and 27% had a cuff pressure above 40 cm H_2_O. Similarly in a prehospital setting Galinski et al. [17] found that among 107 patients, the tracheal tube cuff pressure was larger than 27 cm H_2_O in 79% of the patients. Thus with inflation of air into the cuff until air sealing, the anaesthesiologist has a poor ability to estimate a correct cuff pressure by palpation of the pilot balloon [18,19]. When using inflation of a fixed volume of air into the cuff, a linear relationship between cuff volume and pressure is established [20], but the volume of air required (4.5 ml) to reach 50 cm H_2_O is only 50% larger than that required for establishing the safe tracheal tube cuff pressure of 30 cm H_2_O (3 ml), i.e. the safety margin is low.

Focus on the cuff pressure is often directed to patients exposed to nitrous oxide during anaesthesia. In these patients cuff pressure monitoring with automatic tracheal tube cuff pressure control is both reliable and stable [21] and for paediatric patients intubated with a cuffed tube, the use of a pressure release valve prevents that high tube cuff pressures develop [22,23]. Use of a pressure release valve is relevant especially when a high compliance tracheal tube cuff is used, since it does not prevent a high cuff pressure to be transmitted to epithelia and the thin polyurethane membrane facilitates transmembrane diffusion of nitrous oxide with following rapid increase of the cuff pressure [24].

The window of application of a correct laryngeal mask cuff pressure is broader (< 60 cm H_2_O) and estimation of the cuff pressure by palpation of the pilot balloon is acceptable after some training [25]. Still the use of a cuff pressure gauge is favoured to confirm that a correct cuff pressure is established, both in regard to a tracheal tube and a laryngeal mask, considering the low cost of the device (~100 €).

Among the limitations of this study is it that the cuff pressure was measured only once during anaesthesia and fluctuations in pressure by movement of the patient's head and change in the depth of anaesthesia or the level of neuromuscular blockade are not taken into account. It could be argued that the pressure reported is not representative for the whole period of anaesthesia, but measurements were spread over 358 min to obtain values both at induction of anaesthesia and during surgery. Also we did not register the technique used for inflation of air into the cuff, and we do not know whether the high incidence of cuff pressures above the recommended level is due to that the staff was unaware of the correct cuff pressure, or whether they did not inflate the cuff only to the level that secured the airway. To generalize the results of this study can also be argued: We only included two hospital departments, but a high incidence of cuff pressures above the recommended level is reported by others [16,17,26].

In conclusion this study demonstrates that more than 50% of patients are provided with a too high tracheal tube or laryngeal mask cuff pressure. We consider that to establish the correct cuff pressure is an important element in the anaesthetic procedures and that it involves the determination of the tracheal tube or laryngeal mask cuff pressure, not only at the induction of anaesthesia but also during its maintenance.

## Competing interests

The authors declare that they have no competing interests.

## Authors' contributions

KZR participated in development of the study design, collected the data, and wrote the first draft of the manuscript. NHS conceived of the study and contributed to protocol development and manuscript preparation. AMM developed the study design and contributed to manuscript preparation. HBN performed data analysis and contributed to manuscript preparation. All authors approved the final manuscript.

## Pre-publication history

The pre-publication history for this paper can be accessed here:

http://www.biomedcentral.com/1471-2253/10/20/prepub

## References

[B1] Nordin U The trachea and cuff-induced tracheal injury. An experimental study on causative factors and prevention Acta Otolaryngol Suppl 1977 345 1 71 335778

[B2] Carrol R Hedden M Safar P Intratracheal cuffs: performance characteristics Anesthesiology 1969 31 275 81 5801269

[B3] Seegobin RD van Hasselt Gl Endotracheal cuff pressure and tracheal mucosal blood flow: endoscopic study of effects of four large volume cuffs Br Med J 1984 288 965 8 10.1136/bmj.288.6422.965 PMC1442489 6423162

[B4] Friedrich T Hänsch U Eichfeld U Steinert M Staemmler A Schönfelder M Recurrent laryngeal nerve paralysis as intubation injury? Chirurg 2000 71 539 44 10.1007/s001040051099 10875011

[B5] Wason R Gupta P Gogia AR Bilateral adductor vocal cord paresis following endotracheal intubation for general anaesthesia Anaesth Intensive Care 2004 32 417 8 10.1177/0310057X0403200320 15264741

[B6] Endo K Okabe Y Maruyama Y Tsukatani T Furukawa M Bilateral vocal cord paralysis caused by laryngeal mask airway Am J Otolaryngol 2007 28 126 9 10.1016/j.amjoto.2006.07.001 17362820

[B7] Stewart A Lindsay WA Bilateral hypoglossal nerve injury following the use of the laryngeal mask airway Anaesthesia 2002 57 264 5 10.1046/j.1365-2044.2002.02231.x 11879217

[B8] Brimacombe J Clarke G Keller C Lingual nerve injury associated with the ProSeal laryngeal mask airway: a case report and review of the literature Br J Anaesth 2005 95 420 3 10.1093/bja/aei187 16006489

[B9] Keller C Puehringer F Brimacombe J The influence of cuff volume on oropharyngeal leak pressure and fibreoptic position with the laryngeal mask airway Br J Anaesth 1998 81 186 7 10.1093/bja/81.2.186 9813520

[B10] Brimacombe J Keller C Morris R Mecklem D A comparison of the disposable versus the reusable laryngeal mask airway in paralyzed adult patients Anesth Analg 1998 87 921 4 10.1097/00000539-199810000-00033 9768795

[B11] Burgard G Mollhoff T Prien T The effect of laryngeal mask cuff pressure on postoperative sore throat incidence J Clin Anesth 1996 8 198 201 10.1016/0952-8180(95)00229-4 8703453

[B12] Brimacombe J Holyoake L Keller C Brimacombe N Scully M Barry J Talbutt P Sartain J McMahon P Pharyngolaryngeal neck and jaw discomfort after anaesthesia with the face mask and laryngeal mask airway at high and low cuff volumes in males and females Anesthesiology 2000 93 26 31 10.1097/00000542-200007000-00009 10861142

[B13] Wong JG Heaney M Chambers NA Erb TO von Ungern-Sternberg BS Impact of laryngeal mask airway cuff pressures on the incidence of sore throat in children Paediatr Anaesth 2009 19 464 9 10.1111/j.1460-9592.2009.02968.x 19281479

[B14] Wajed SA Streets CG Bremner CG DeMeester TR Elevated body mass disrupts the barrier to gastroesophageal reflux Arch Surg 2001 136 1014 8 10.1001/archsurg.136.9.1014 11529823

[B15] Pelosi P Croci M Ravagnan I Tredici S Pedoto A Lissoni A Gattinoni L The effects of body mass on lung volumes, respiratory mechanics, and gas exchange during general anaesthesia Anesth Analg 1998 87 654 60 10.1097/00000539-199809000-00031 9728848

[B16] Sengupta P Sessler DI Maglinger P Wells S Vogt A Durrani J Wadhwa A Endotracheal tube cuff pressure in three hospitals, and the volume required to produce an appropriate cuff pressure BMC Anesthesiol 2004 4 8 10.1186/1471-2253-4-8 PMC535565 15569386

[B17] Galinski M Tréoux V Garrigue B Lapostolle F Borron SW Adnet F Intracuff pressures of endotracheal tubes in the management of airway emergencies: the need for pressure monitoring Ann Emerg Med 2006 47 545 7 10.1016/j.annemergmed.2005.08.012 16713783

[B18] Hoffman RJ Parwani V Hahn IH Experienced emergency medicine physicians cannot safely inflate or estimate endotracheal tube cuff pressure using standard techniques Am J Emerg Med 2006 24 139 43 10.1016/j.ajem.2005.07.016 16490640

[B19] Stewart SL Secrest JA Norwood BR Zachary R A comparison of endotracheal tube cuff pressures using estimation techniques and direct intracuff measurement AANA J 2003 71 443 7 15098531

[B20] Hoffman RJ Dahlen JR Lipovic D Stürmann KM Linear Correlation of Endotracheal Tube Cuff Pressure and Volume West J Emerg Med 2009 10 137 9 PMC2729210 19718371

[B21] Kunitz O Jansen R Ohnsorge E Haaf-vonBelow S Schulz-Stübner S Rossaint R Cuff pressure monitoring and regulation in adults Anaesthesist 2004 53 334 40 10.1007/s00101-004-0664-6 15042308

[B22] Dullenkopf A Bernet-Buettiker V Maino P Weiss M Performance of a novel pressure release valve for cuff pressure control in pediatric tracheal tubes Paediatr Anaesth 2006 16 19 24 10.1111/j.1460-9592.2005.01686.x 16409524

[B23] Weiss M Dullenkopf A Fischer JE Keller C Gerber AC Prospective randomized controlled multi-centre trial of cuffed or uncuffed endotracheal tubes in small children Br J Anaesth 2009 103 867 73 10.1093/bja/aep290 19887533

[B24] Dullenkopf A Gerber AC Weiss M Nitrous oxide diffusion into tracheal tube cuffs: comparison of five different tracheal tube cuffs Acta Anaesthesiol Scand 2004 48 1180 4 10.1111/j.1399-6576.2004.00483.x 15352966

[B25] Keller C Brimacombe JR Laryngeal mask airway intracuff pressure estimation by digital palpation of the pilot balloon: a comparison of reusable and disposable masks Anaesthesia 1999 54 183 186 10.1046/j.1365-2044.1999.00664.x 10215716

[B26] Braz JR Navarro LH Takata IH Nascimento Júnior P Endotracheal tube cuff pressure: need for precise measurement Sao Paulo Med J 1999 117 243 7 10.1590/s1516-31801999000600004 10625887

